# Recovery of Fatty Acid and Volatile Flavor Compound Composition in Farmed Tiger Puffer (*Takifugu rubripes*) with a Fish Oil-Finishing Strategy

**DOI:** 10.3390/md21020122

**Published:** 2023-02-13

**Authors:** Lin Li, Feiran Zhang, Xiaoxue Meng, Xishuai Cui, Qiang Ma, Yuliang Wei, Mengqing Liang, Houguo Xu

**Affiliations:** 1 Yellow Sea Fisheries Research Institute, Chinese Academy of Fishery Sciences, Qingdao 266071, China; 2 Laboratory for Marine Fisheries Science and Food Production Processes, Qingdao National Laboratory for Marine Science and Technology, Qingdao 266237, China; 3 College of Fisheries and Life Sciences, Shanghai Ocean University, Shanghai 201306, China

**Keywords:** long-chain polyunsaturated fatty acid, muscle flavor, DHA recovery, feeding strategy

## Abstract

Booming fish farming results in a relative shortage of fish oil (FO) supply, meaning that alternative oils are increasingly used in fish feeds, which leads to reduction of long-chain polyunsaturated fatty acids (LC-PUFAs) and other relevant changes in fish products. This study investigated the efficacy of an FO-finishing strategy in recovering the muscle quality of farmed tiger puffer. An eight-week feeding trial (growing-out period) was conducted with five experimental diets, in which graded levels (0 (control), 25, 50, 75, and 100%) of added FO were replaced by poultry oil (PO). Following the growing-out period was a four-week FO-finishing period, during which fish in all groups were fed the control diet. Dietary PO significantly decreased the muscle LC-PUFA content, whereas in general, the FO-finishing strategy recovered it to a level comparable with that of the group fed FO continuously. The recovery efficiency of EPA was higher than that of DHA. Dietary PO also led to changes of volatile flavor compounds in the muscle, such as butanol, pentenal, and hexenal, whereas the FO-finishing strategy mitigated the changes. In conclusion, the FO-finishing strategy is promising in recovering the LC-PUFA and volatile-flavor-compound composition in farmed tiger puffer after the feeding of PO-based diets.

## 1. Introduction

Fish are the main source of n-3 long-chain polyunsaturated fatty acids (LC-PUFAs), mainly 22:6n-3 (docosahexaenoic acid, DHA) and 20:5n-3 (eicosapentaenoic acid, EPA) [1]. Aquaculture satisfies the growing global demand for fish, but also consumes an increasing share of the world’s wild-fish fishery via the use of fishmeal and fish oil (FO) in fish feeds [2]. Therefore, increasing levels of alternative sources such as plant ingredients, livestock processing by-products, and single-cell materials are being used in fish feeds.

Poultry oil (PO) is a by-product of chicken processing and has a relatively low price and a large annual production. With high contents of 16:0, 18:1n-9, and 18:2n-6, PO is a potential lipid source for fish feeds. Partial or complete FO replacement by PO has proved feasible in diets of many aquaculture fish species [3,4,5,6,7,8,9,10,11,12]. It has been widely accepted that the fatty-acid profile of farmed fish generally reflects that of their diets [13]. Therefore, as expected, dietary replacement of FO by PO in the aforementioned studies led to decreased LC-PUFA content in the fish.

The FO-finishing strategy, namely, re-feeding FO for a period after long-term feeding with alternative oils, has been demonstrated to be an efficient way to restore the LC-PUFA content in farmed fish when alternative oils are used to replace FO in fish feeds [13]. This strategy has been practiced in sunshine bass (*Morone chrysops* × *M. saxatilis*) [14] and rainbow trout (*Oncorhynchus mykiss*) [15] when PO is used in the feeds, but the LC-PUFA recovery efficiency is not consistent between species. Moreover, the effects of dietary PO on the volatile-flavor-compound composition in farmed fish, as well as their response to FO-finishing, have not been studied.

Volatile flavor compounds, which consist of aldehyde, alcohol, acid, ketone, phenol, and heterocyclic compounds containing nitrogen and sulfur, are an important component of fish flavor. Polyunsaturated fatty acids are major substrates of volatile-flavor-compound formation. This has been demonstrated in a number of fish species such as crucian carp (*Carassius auratus gibelio*), Nile tilapia (*Oreochromis niloticus*), gilthead seabream (*Sparus aurata*), tench (*Tinca tinca*), brown trout (*Salmo trutta*), turbot (*Psetta maxima*), rainbow trout, and Senegal sole (*Solea senegalensis*) [16,17,18,19,20,21,22,23]. These studies also suggest that the dietary lipid source significantly affects the muscle-volatile-flavor-compound composition via changes in fatty-acid metabolism.

With these considerations in mind, the present study was aimed at comprehensively evaluating the efficacy of FO-finishing in recovering LC-PUFA and volatile-flavor-compound composition in farmed tiger puffer, which is an important aquaculture species in Asia [24]. Results of this study will provide new insights into the manipulation of fish-product quality via the feeding strategy.

## 2. Results

### 2.1. Fatty-Acid Composition in Muscle and Liver

**Figure 1:**
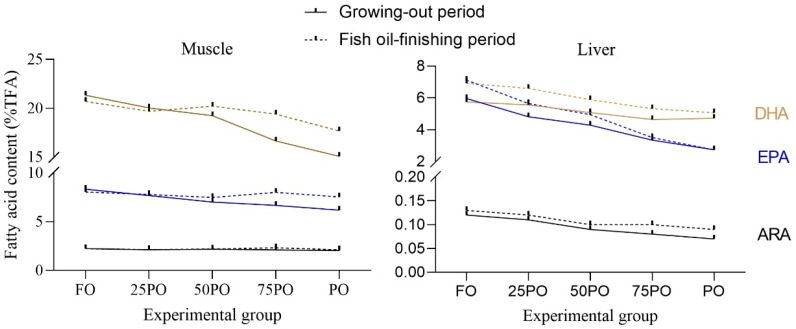
LC-PUFA contents in the muscle and liver of experimental tiger puffer.

Fish grew normally during the whole experiment [25]. The average fish body weight at the end of the growing-out period and FO-finishing period was 41.99 g and 82.37 g, respectively. At the end of the growing-out period, with increasing levels of dietary PO, the contents of monounsaturated fatty acid (MUFA) and n-6 PUFA in the muscle significantly (*p* < 0.05) increased; that of n-3 PUFA significantly decreased; but the content of saturated fatty acid (SFA) was stable (Table 1, Figure 1). In particular, the DHA content decreased from 21.32% TFA in the FO group to 15.06% in the PO group (71% of that in the FO group), and accordingly, the EPA content decreased from 8.32% to 6.18% (74% of that in the FO group). However, the 22:5n-3 content slightly increased from the FO group to the PO group. After FO-finishing, the change in MUFA and PUFA caused by PO was substantially mitigated (Table 2, Figure 1). The DHA and EPA was recovered to be 85% and 94% of that in the control group continuously fed FO, respectively.

The liver fatty-acid composition showed a similar trend in response to PO supplementation, but the change was more drastic compared to the muscle except that the n-6 PUFA was stable among different groups (Table 3, Figure 1). In particular, at the end of the growing-out period, the DHA and EPA in the PO group was only 38% and 46% of that in the FO control group, respectively. Similar mitigating effects of the FO-finishing strategy were observed in the liver (Table 4, Figure 1). After FO-finishing, the DHA and EPA in the PO group was recovered to be 73% and 82% of that in the FO control group, respectively.

### 2.2. Volatile Flavor Components in the Muscle

**Figure 2:**
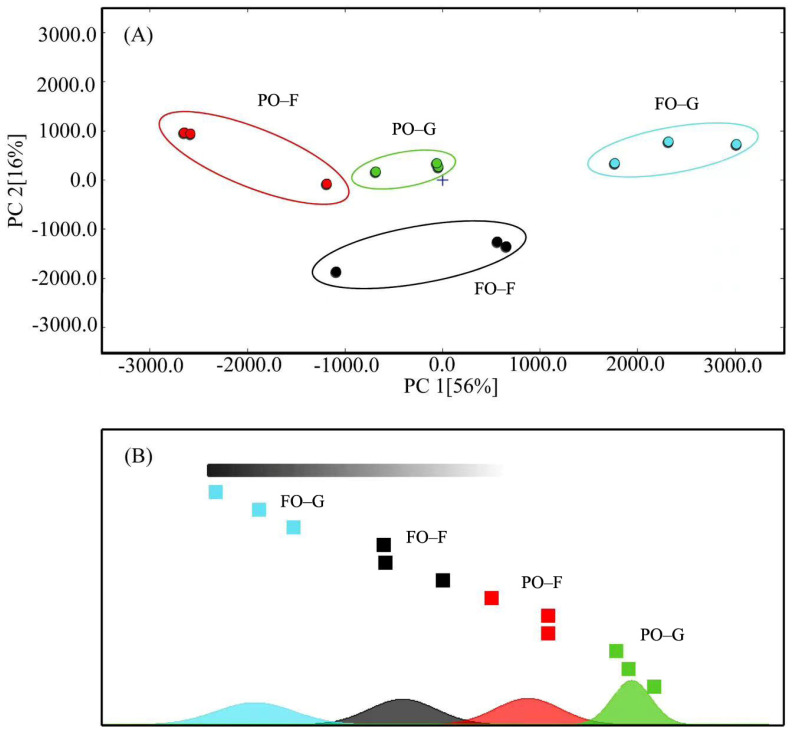
Principal component analysis (PCA) (**A**) and Euclidean distance (**B**) of volatile flavor compounds in the muscle. FO–G and PO–G: the fish oil and poultry oil group at the end of growing-out period, respectively; FO–F and PO–F: the fish oil and poultry oil group after fish oil-finishing, respectively.

**Figure 3:**
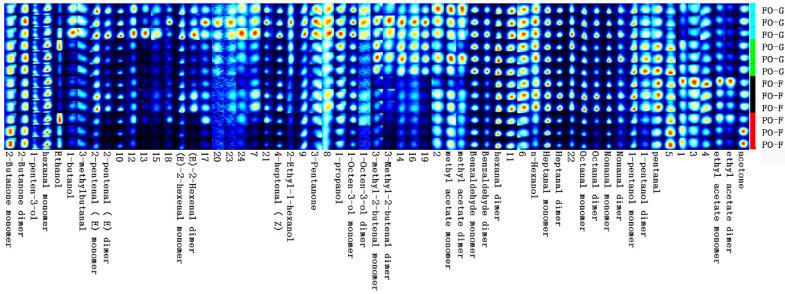
Gallery plot of the volatile flavor compounds in the muscle. The brightness indicates relative compound abundance. A column represents the signal peak of a certain volatile organic compound in different samples. A line represents all signal peaks of volatile organic compound selected from a certain sample. Compounds named as numbers were not successfully identified. The meanings of FO–G, PO–G, FO–F, and PO–F are the same as in Figure 3.

From all muscle samples, a total of 61 volatile flavor components were detected, of which 37 were successfully identified (Figure 2 and Figure 3, Table S1). Most of these compounds were small-molecular alcohols, ketones, or aldehydes. At the end of the growing-out period, the PO group had lower abundance of 1-butanol, 3-methyl butanol, 2-pentenal (E) dimer, (E)-2-hexenal monomer, (E)-2-hexenal dimer, 4-heptenal (Z), 3-pentanone, 1-octen-3-ol monomer, 1-octen-3-ol dimer, and octanal dimer, but higher abundance of ethanol, 1-pentanol dimer, and pentanal than the FO control group. At the end of the FO-finishing period, the difference between the PO and FO groups was smaller, but obvious differences in ethanol, 2-pentenal (E) dimer, (E)-2-hexenal monomer, (E)-2-hexenal monomer, (E)-2-hexenal dimer, 4-heptenal (Z), and octanal dimer could still be observed.

### 2.3. Determination of Muscle Odor with Electronic Nose

In general, the PCA (Figure S1) and LDA (Figure S2) in the muscle-odor analysis with electronic nose showed that at the end of the growing-out period, there was large distance between groups FO and PO, but this distance was substantially shortened at the end of the FO-finishing period.

## 3. Discussion

Poultry oil (PO) is increasingly used in fish feeds. However, a major concern of human consumers when fish oil (FO) is replaced by PO is the reduction of LC-PUFA in fish products. In the current study, increasing levels of dietary PO linearly decreased the n-3 LC-PUFA content in fish. At the end of the growing-out period, the muscle DHA content in the PO group was 70.6% of that in the FO control group. This ratio was higher than that observed in other fish species, namely, yellowtail kingfish (*Seriola lalandi*), 49.7% [6]; largemouth bass (*Micropterus salmoides*), 56.5% [7]; and European seabass (*Dicentrarchus labrax*), 30.5% [11]. The discrepancy could be mainly related to the fish species and most importantly, feeding duration. A longer period of PO feeding (12 weeks for largemouth bass; 16 weeks for European seabass) resulted in lower DHA content in fish muscle.

After FO-finishing, the DHA content in group PO was restored to be 85% of that in the FO control group. In most similar studies, an FO-finishing diet was able to restore the DHA content to between 70 and 90% of that in fish fed FO continuously [13]. However, in a study with smaller rainbow trout (initial body weight, 1.4 g), a 28-day FO-finishing period after 80 days of a growing-out feeding trial with PO completely restored the muscle DHA and EPA to levels comparable to those achieved via the continuous use of an FO-based diet [15]. Fish size is a clear determinant in the rate of fatty-acid turnover. Smaller fish have a higher rate of fatty-acid turnover and thus have higher capacity for restoring LC-PUFA. In another study on FO-finishing after PO feeding (50% FO replaced), 56 days of FO-finishing after 140 days of growing-out restored the muscle DHA, EPA, and ARA in sunshine bass to be 94%, 92%, and 100% of that in the FO control group, respectively [14]. These ratios were higher than those in the present study. It is clear that the duration of the finishing period is one of the most important factors influencing the final muscle LC-PUFA content. A longer finishing period resulted in higher restoration ratio of muscle LC-PUFA.

There are many studies regarding the use of an FO-finishing strategy after feeding diets based on other alternative oils such as rapeseed oil, palm oil, soybean oil, linseed oil, coconut oil, sunflower oil, and olive oil [14,26,27,28,29,30]. Overall, the effect of alternative oil type on the efficiency of LC-PUFA recovery during the subsequent FO-finishing period appears to be complicated and species-specific. Relevant results in farmed fish species have been summarized in a recent review article [13]. In other marine fish species such as gilthead sea bream (*Sparus aurata*) and European sea bass, differences were observed in the degree of LC-PUFA recovery during the FO-finishing period when different alternative lipid sources such as rapeseed oil, soybean oil, and linseed oil were previously fed to fish [26,27,28]. Specific to tiger puffer, a previous study revealed that washing-out the PO in the muscle was easier compared to soybean oil, but more difficult compared to beef tallow [24]. In the liver, however, washing-out the PO was easier compared to rapeseed oil, but also more difficult compared to beef tallow. In another study regarding washing-out PO, it was found that washing-out the PO in sunshine bass was more difficult compared to washing-out grape oil and linseed oil [14]. The LC-PUFA restoration efficiency could be closely related to the efficiency of washing-out the characteristic fatty acids in different terrestrially-sourced oils. However, to date, no clear conclusion has been made by previous studies regarding the difference in washing-out efficiency among different individual fatty acids or different fatty-acid types. In addition, different terrestrially-sourced oils may lead to different lipid deposition levels in fish body, which could also affect the LC-PUFA restoration efficiency.

For tiger puffer, liver is also an edible part, particularly considering that nowadays tetrodotoxin is nearly undetectable in farmed fish [31]. Therefore, the restoration of LC-PUFA in the liver should also not be neglected when the FO-finishing strategy is practiced. Compared to muscle, the LC-PUFA restoration efficiency in the liver after FO-finishing was lower. This may be due to the fact that a limited number of fatty acids, such as DHA, EPA, and 16:0, tend to be selectively incorporated into muscle which is rich in polar lipids [32]. In other tissues with conservative fatty-acid profiles such as the neural tissue, in which LC-PUFA is vital for chemical communication/homeostasis, the LC-PUFA restoration efficiency could also be high, although no tissues other than muscle and liver were analyzed in this study.

Regarding the difference in efficiency of LC-PUFA restoration among individual fatty acids, EPA showed a higher efficiency than DHA in both muscle and liver of tiger puffer. This was different from most other similar studies, which showed that DHA is generally more completely restored, compared to EPA [13]. The present result could be related to the fact that the EPA content in tiger puffer is much lower than DHA, and thus is easier to be restored.

In addition to fatty-acid composition, the volatile-flavor-compound compositions also largely contribute to the fish-fillet quality. The identified volatile flavor compound in tiger puffer mainly consists of aldehyde, alcohol, ketone, phenol, and heterocyclic compounds containing nitrogen and sulfur. The volatile flavor compounds differentially abundant between the FO control group and the PO group were mainly flavor aldehydes and alcohols. Aldehydes have a lower odor threshold and a greater flavor effect on aquatic products, but alcohols have a higher odor threshold and a smaller contribution to flavor.

Many of the flavor aldehydes such as heptanal, octanal, and nonanal are products of the oxidation of oleic and linoleic acids [33,34,35,36]. At the end of the growing-out period, the volatile flavor compound profile was clearly different between the FO and PO groups. The high abundance of 1-pentanol dimer in the PO group may result in the flavors of mushroom, earth, and wax, which are generally considered “off tastes” in sensory-panel testing. The PO group also had higher abundance of pentanal, which could be derived from the oxidation of n-6 PUFA, and has almond, pungent, and malty flavors [35]. In contrast, in the muscle of the FO control group, the higher abundance of butanol, 2-pentenal, (E)-2-hexenal, and 4-heptenal (Z) would generate more flavor of butter, fruit (apple, orange, banana), cheese, boiled potato, and cooked fish. These compounds could also make the fillets taste sweeter and fattier. The adverse effects of PO supplementation on the volatile-flavor-compound profile of muscle were largely, but not completely, mitigated by the FO-finishing. At the end of the FO-finishing period, obvious differences in ethanol, 2-pentenal (E) dimer, (E)-2-hexenal monomer, (E)-2-hexenal monomer, (E)-2-hexenal dimer, 4-heptenal (Z), and octanal dimer could still be observed. In particular, after FO finishing, the PO group still had a lower abundance of heptanal, octanal, and hexanol than the FO control group. Heptanal and octanal usually have fatty, dry fish, grass, and fruit flavors. Hexanol arises from lipoxygenase and hydroperoxide-lyase activities and has the taste of grass [37]. Lower abundance of these compounds indicates that the muscle from the PO group may have had less fishy flavor but also less grass flavor. In addition, for the FO control group, the abundance of many volatile compounds at week 8 was lower than that at week 12, indicating the decrease of volatile compounds with fish growth. Similar results were found in Atlantic salmon (*Salmo salar*) [38].

## 4. Materials and Methods

### 4.1. Experimental Diets

Five experimental diets were formulated. Fish oil (FO) was used as the sole added oil in the control diet. In other diets, added FO was replaced by poultry oil (PO, refined from duck skin, Shandong Haiding Agriculture and Animal Husbandry Co., Ltd., Jinan, China) at different levels, namely, 25%, 50%, 75%, and 100%. The five experimental diets were designated as FO (control), 25PO, 50PO, 75PO, and PO, respectively (Table 5 and Table 6). The pellet feeds with a diameter of 2 mm were made with a laboratory-level single-screw pelleting machine and dried at 55 °C. The experimental diets were stored at −20 °C prior to use. The proximate composition analysis of experimental diets was performed according to the standard methods of the Association of Official Analytical Chemists (AOAC). In brief, the moisture content was measured by drying the samples to a constant weight at 105 °C; the protein content was assayed by measuring nitrogen content (N × 6:25) using the Kjeldahl method; the lipid content was assayed with petroleum ether extraction using the Soxhlet method; and the ash content was measured by incineration in a muffle furnace at 550 °C for 8 h.

### 4.2. Feeding Procedure and Sampling

**Figure 4:**
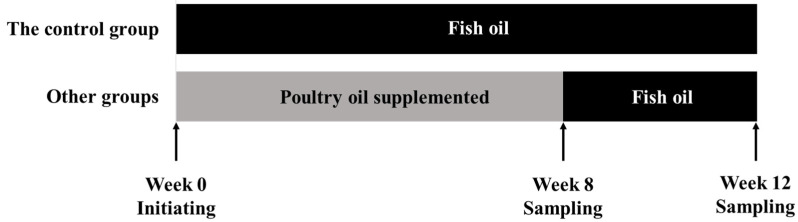
Experimental design and feeding periods.

Tiger puffer juveniles (average initial body weight, 12.3 ± 0.5 g; average body length, 6.5 ± 0.5 cm) were purchased from Hongqi Modern Fishery Industrial Park (Rizhao, China), and transported to the Yellow Sea Aquaculture Co., Ltd. (Yantai, China), where the feeding trial was conducted. At the beginning of the experiment, 600 randomly selected healthy fish were divided into 15 polyethylene tanks (0.7 × 0.7 × 0.4 m). Each tank was stocked with 40 fish. Each diet was randomly fed to triplicate tanks. Fish were hand-fed to apparent satiation three times daily (7:00, 12:00, and 18:00). The feeding trial lasted 12 weeks (Figure 4). Following the first 8-week growing-out period, during which the five experiment diets were normally fed to experimental fish, was a four-week FO-finishing period, during which fish in all five groups were fed the FO control diet.

Sampling was conducted at the end of both the growing-out period (week 8) and the FO-finishing period (week 12). Before sampling, fish were fasted for 24 h. In each sampling point, four fish were randomly selected from each tank, and the muscle and liver samples were collected. The samples were immediately frozen with liquid nitrogen and then stored at −76 °C before use. All sampling protocols, as well as all fish rearing practices, were reviewed and approved by the Animal Care and Use Committee of Yellow Sea Fisheries Research Institute.

### 4.3. Analysis of Fatty-Acid Composition

The fatty-acid compositions of oils, diets, muscles, and livers were analyzed with gas chromatography, as previously described [39]. Lipids in the samples were firstly extracted with the chloroform methanol method. Fatty acids in the extracted oil were then saponified and methylated with KOH-methanol and HCL-methanol. Gas chromatograph (GC-2010 pro, Shimadzu, Kyoto, Japan) equipped with a quartz capillary column (SH-RT-2560, 100 m × 0.25 mm × 0.20 um) and a flame ionization detector was used in the analysis. The results are expressed as percentage of each fatty acid relative to the total fatty acids (%TFA).

### 4.4. Analysis of Volatile Organic Compounds in the Muscle

Two typical groups, FO and PO, was subjected to the analysis of volatile organic compounds in the muscle, which was conducted with gas chromatography−ion migration spectrometry (GC-IMS). A FlavourSpec^®^ (G.A.S, Dordmund, Germany) platform equipped with a MXT-5 column (RESTEK, Bellefonte, PA, USA; 15 m × 0.53 mm × 1.0 μm) was used in this analysis. The column and IMS temperatures were 60 and 45 °C, respectively. High-purity nitrogen (purity, 99.999%) was used as the carrier gas. Three grams muscle samples were accurately weighed and placed in a 20 mL vial. The samples were incubated at 60 °C (500 r/min) for 15 min. The injection volume was 500 μL, and the temperature of the automatic injection needle was 85 °C. Built-in software VOCal and three tools including Reporter, Gallery Plot, and Dynamic PCA were used in the result report.

### 4.5. Electronic-Nose Analysis

The odor analysis for the muscle samples from groups FO and PO was performed with a PEN-3 portable electronic nose (Airsense, Schwerin, Germany). One gram of muscle sample was weighed and placed it in a 20 mL vial with lid and sealed. The samples were then heated at 50 °C in a water bath for 10 min, and finally subjected to electronic-nose analysis. Winmuster software was used for data collection and processing, generating results of principal component analysis (PCA) and linear discrimination method (LDA). Ten different metal-oxide sensors (W1C, benzene; W5S, nitrogen oxide; W3C, amine; W6S, hydride; W5C, short alkane; W1S, methyl; W1W, inorganic sulfide; W2S, alcohols; W2W, organic sulfide; and W3S, long chain alkanes) were used to detect the main volatile compounds in the samples.

### 4.6. Statistical Analyses

All data were analyzed by one-way ANOVA in SPSS 16.0. Multiple comparisons were performed using Tukey’s test, and the significance level was decided when *p* < 0.05. The results are expressed as mean ± standard error.

## 5. Conclusions

In conclusion, results of this study suggested that the adverse effects of dietary poultry oil on fatty acid and volatile flavor compound compositions of farmed tiger puffer can be largely mitigated by a fish-oil-finishing strategy. However, despite the high efficiency of the fish-oil-finishing strategy in this study, the efficiency when there is a very long period of poultry-oil feeding remains unknown and warrants further study.

## Figures and Tables

**Table 1:** Fatty-acid compositions in the muscle at the end of the growing-out period (%TFA, mean ± standard error).

Fatty Acid	FO	25PO	50PO	75PO	PO
14:0	0.67 ± 0.05 ^c^	0.56 ± 0.05 ^bc^	0.50 ± 0.01 ^abc^	0.42 ± 0.03 ^ab^	0.36 ± 0.03 ^a^
16:0	23.22 ± 0.25	22.79 ± 0.31	22.98 ± 0.06	22.54 ± 0.10	22.54 ± 0.25
18:0	12.39 ± 0.15	12.51 ± 0.18	12.13 ± 0.05	12.12 ± 0.28	11.92 ± 0.13
20:0	0.35 ± 0.15	0.42 ± 0.00	0.32 ± 0.00	0.25 ± 0.01	0.14 ± 0.03
∑SFA	36.63 ± 0.54 ^b^	36.28 ± 0.18 ^ab^	35.92 ± 0.09 ^ab^	35.33 ± 0.34 ^ab^	34.96 ± 0.20 ^a^
16:1n-7	1.12 ± 0.13	1.02 ± 0.08	0.93 ± 0.04	0.90 ± 0.05	0.86 ± 0.04
17:1n-7	0.53 ± 0.02 ^ab^	0.55 ± 0.03 ^b^	0.47 ± 0.01 ^ab^	0.45 ± 0.01 ^ab^	0.44 ± 0.04 ^a^
18:1n-9	11.15 ± 0.18 ^a^	12.57 ± 0.08 ^b^	13.94 ± 0.11 ^c^	15.19 ± 0.11 ^d^	16.34 ± 0.31 ^e^
24:1n-9	0.23 ± 0.05	0.41 ± 0.05	0.24 ± 0.08	0.72 ± 0.35	0.31 ± 0.11
∑MUFA	13.02 ± 0.33 ^a^	14.54 ± 0.19 ^b^	15.57 ± 0.17 ^b^	17.27 ± 0.27 ^c^	17.95 ± 0.47 ^c^
18:2n-6	10.82 ± 0.22 ^a^	12.13 ± 0.30 ^b^	12.86 ± 0.09 ^b^	14.57 ± 0.20 ^c^	16.04 ± 0.11 ^d^
20:2n-6	0.68 ± 0.03 ^a^	0.77 ± 0.01 ^b^	0.78 ± 0.01 ^b^	0.83 ± 0.02 ^bc^	0.88 ± 0.01 ^c^
20:4n-6	2.23 ± 0.08	2.12 ± 0.07	2.18 ± 0.04	2.10 ± 0.02	2.02 ± 0.04
∑n-6 PUFA	13.73 ± 0.17 ^a^	15.01 ± 0.30 ^b^	15.82 ± 0.08 ^b^	17.50 ± 0.19 ^c^	18.94 ± 0.13 ^d^
18:3n-3	0.28 ± 0.05	0.24 ± 0.03	0.26 ± 0.00	0.24 ± 0.02	0.20 ± 0.01
20:5n-3	8.32 ± 0.04 ^d^	7.67 ± 0.11 ^c^	7.00 ± 0.02 ^b^	6.66 ± 0.10 ^ab^	6.18 ± 0.23 ^a^
22:5n-3	4.89 ± 0.15	4.86 ± 0.05	4.81 ± 0.07	5.11 ± 0.13	5.22 ± 0.09
22:6n-3	21.32 ± 0.73 ^b^	20.01 ± 0.59 ^b^	19.22 ± 0.14 ^b^	16.63 ± 0.44 ^a^	15.06 ± 0.23 ^a^
∑n-3 PUFA	34.81 ± 0.81 ^c^	32.79 ± 0.53 ^bc^	31.3 ± 0.06 ^b^	28.64 ± 0.20 ^a^	26.65 ± 0.54 ^a^
∑n-3/∑n-6	2.54 ± 0.08 ^c^	2.19 ± 0.07 ^b^	1.98 ± 0.01 ^b^	1.64 ± 0.03 ^a^	1.41 ± 0.02 ^a^

Data in a same row not sharing a same superscript letter are significantly different (*p* ˂ 0.05). TFA: total fatty acid; SFA: saturated fatty acid; MUFA: monounsaturated fatty acid; n-6 PUFA: n-6 poly-unsaturated fatty acid; n-3 PUFA: n-3 polyunsaturated fatty acid.

**Table 2:** Fatty-acid compositions in the muscle after fish oil-finishing (%TFA, mean ± standard error).

Fatty Acid	FO	25PO	50PO	75PO	PO
14:0	0.75 ± 0.02	0.80 ± 0.09	0.67 ± 0.02	0.62 ± 0.03	0.65 ± 0.01
16:0	23.39 ± 0.20 ^b^	22.47 ± 0.39 ^ab^	22.3 ± 0.30 ^ab^	22.38 ± 0.14 ^ab^	22.04 ± 0.19 ^a^
18:0	12.61 ± 0.30	12.52 ± 0.44	12.80 ± 0.45	12.46 ± 0.13	12.73 ± 0.35
20:0	0.53 ± 0.01	0.54 ± 0.03	0.52 ± 0.06	0.44 ± 0.01	0.44 ± 0.02
∑SFA	37.28 ± 0.47	36.33 ± 0.74	36.28 ± 0.76	35.89 ± 0.10	35.86 ± 0.38
16:1n-7	1.11 ± 0.02	1.23 ± 0.12	1.05 ± 0.03	0.97 ± 0.06	0.99 ± 0.03
17:1n-7	0.63 ± 0.06	0.59 ± 0.03	0.63 ± 0.04	0.54 ± 0.00	0.55 ± 0.04
18:1n-9	10.69 ± 0.15 ^a^	11.43 ± 0.26 ^ab^	11.51 ± 0.25 ^abc^	11.64 ± 0.08 ^bc^	12.35 ± 0.11 ^c^
24:1n-9	0.29 ± 0.03	0.34 ± 0.03	0.40 ± 0.02	0.37 ± 0.03	0.50 ± 0.09
∑MUFA	12.72 ± 0.19 ^a^	13.59 ± 0.38 ^ab^	13.59 ± 0.26 ^ab^	13.52 ± 0.06 ^ab^	14.39 ± 0.16 ^b^
18:2n-6	10.81 ± 0.23 ^a^	11.37 ± 0.09 ^a^	11.54 ± 0.13 ^ab^	12.19 ± 0.24 ^bc^	12.79 ± 0.10 ^c^
20:2n-6	0.70 ± 0.04 ^a^	0.87 ± 0.04 ^b^	0.85 ± 0.01 ^b^	0.90 ± 0.03 ^b^	0.92 ± 0.03 ^b^
20:4n-6	2.23 ± 0.02	2.13 ± 0.10	2.19 ± 0.08	2.32 ± 0.06	2.12 ± 0.04
∑n-6 PUFA	13.74 ± 0.24 ^a^	14.37 ± 0.02 ^ab^	14.57 ± 0.07 ^b^	15.41 ± 0.21 ^c^	15.83 ± 0.16 ^c^
18:3n-3	0.11 ± 0.11	0.29 ± 0.07	0.16 ± 0.08	0.21 ± 0.05	0.15 ± 0.08
20:5n-3	8.02 ± 0.29	7.78 ± 0.28	7.46 ± 0.24	7.99 ± 0.05	7.52 ± 0.17
22:5n-3	5.26 ± 0.10 ^a^	5.63 ± 0.18 ^ab^	5.71 ± 0.06 ^ab^	5.63 ± 0.16 ^ab^	6.02 ± 0.10 ^b^
22:6n-3	20.69 ± 0.32 ^b^	19.68 ± 0.42 ^b^	20.19 ± 0.71 ^b^	19.38 ± 0.20 ^ab^	17.65 ± 0.23 ^a^
∑n-3 PUFA	34.08 ± 0.43 ^b^	33.38 ± 0.49 ^ab^	33.52 ± 0.93 ^ab^	33.22 ± 0.09 ^ab^	31.33 ± 0.54 ^a^
∑n-3/∑n-6	2.48 ± 0.04 ^c^	2.32 ± 0.03 ^bc^	2.30 ± 0.06 ^bc^	2.16 ± 0.04 ^ab^	1.98 ± 0.02 ^a^

Data in a same row not sharing a same superscript letter are significantly different (*p* ˂ 0.05). TFA: total fatty acid; SFA: saturated fatty acid; MUFA: monounsaturated fatty acid; n-6 PUFA: n-6 poly-unsaturated fatty acid; n-3 PUFA: n-3 polyunsaturated fatty acid. Data in a same row not sharing a same superscript letter are significantly different (*p* ˂ 0.05).

**Table 3:** Fatty-acid compositions in the liver at the end of the growing-out period (%TFA, mean ± standard error).

Fatty Acid	FO	25PO	50PO	75PO	PO
14:0	3.49 ± 0.09 ^c^	3.20 ± 0.08 ^c^	2.55 ± 0.02 ^b^	2.29 ± 0.05 ^b^	1.84 ± 0.02 ^a^
16:0	19.51 ± 0.63	20.65 ± 0.24	19.15 ± 0.26	19.93 ± 0.38	19.58 ± 0.13
18:0	8.63 ± 0.76	9.47 ± 0.48	7.91 ± 0.19	9.14 ± 0.75	8.68 ± 0.63
20:0	0.52 ± 0.03 ^c^	0.48 ± 0.01 ^c^	0.35 ± 0.02 ^b^	0.31 ± 0.01 ^ab^	0.24 ± 0.01 ^a^
∑SFA	32.15 ± 1.32	33.80 ± 0.66	29.96 ± 0.25	31.66 ± 1.03	30.34 ± 0.60
16:1n-7	8.53 ± 0.21 ^b^	8.31 ± 0.23 ^ab^	8.41 ± 0.12 ^ab^	7.15 ± 0.40 ^a^	7.32 ± 0.35 ^ab^
18:1n-9	23.90 ± 0.18 ^a^	26.54 ± 0.46 ^b^	30.83 ± 0.47 ^c^	34.43 ± 0.26 ^d^	37.25 ± 0.57 ^e^
20:1n-9	2.22 ± 0.22	2.03 ± 0.22	2.39 ± 0.04	1.98 ± 0.17	2.01 ± 0.08
22:1n-9	0.25 ± 0.00 ^d^	0.24 ± 0.01 ^cd^	0.21 ± 0.01 ^bc^	0.18 ± 0.00 ^ab^	0.17 ± 0.01 ^a^
∑MUFA	34.90 ± 0.31 ^a^	37.12 ± 0.63 ^b^	41.85 ± 0.30 ^c^	43.74 ± 0.51 ^c^	46.74 ± 0.37 ^d^
18:2n-6	12.26 ± 0.28	12.27 ± 0.12	12.84 ± 0.19	12.58 ± 0.35	12.74 ± 0.25
18:3n-6	0.19 ± 0.01 ^d^	0.17 ± 0.00 ^cd^	0.15 ± 0.00 ^bc^	0.13 ± 0.01 ^ab^	0.13 ± 0.01 ^a^
20:2n-6	0.79 ± 0.03	0.83 ± 0.03	0.84 ± 0.03	0.76 ± 0.02	0.78 ± 0.05
20:4n-6	0.12 ± 0.00 ^b^	0.11 ± 0.00 ^b^	0.09 ± 0.01 ^a^	0.08 ± 0.01 ^a^	0.07 ± 0.01 ^a^
∑n-6 PUFA	13.35 ± 0.31	13.37 ± 0.14	13.91 ± 0.23	13.54 ± 0.37	13.72 ± 0.31
20:3n-3	0.58 ± 0.03 ^b^	0.58 ± 0.05 ^b^	0.51 ± 0.01 ^ab^	0.46 ± 0.03 ^ab^	0.42 ± 0.01 ^a^
20:5n-3	5.96 ± 0.25 ^c^	4.82 ± 0.11 ^b^	4.29 ± 0.14 ^b^	3.35 ± 0.23 ^a^	2.74 ± 0.07 ^a^
22:5n-3	4.14 ± 0.13 ^c^	3.48 ± 0.08 ^b^	3.42 ± 0.14 ^b^	2.85 ± 0.02 ^a^	2.45 ± 0.03 ^a^
22:6n-3	7.12 ± 0.31 ^c^	5.65 ± 0.04 ^b^	4.95 ± 0.23 ^b^	3.51 ± 0.04 ^a^	2.73 ± 0.06 ^a^
∑n-3 PUFA	17.78 ± 0.69 ^d^	14.52 ± 0.25 ^c^	13.17 ± 0.33 ^c^	10.17 ± 0.23 ^b^	8.34 ± 0.12 ^a^
∑n-3/∑n-6	1.33 ± 0.02 ^e^	1.09 ± 0.01 ^d^	0.95 ± 0.03 ^c^	0.75 ± 0.01 ^b^	0.61 ± 0.01 ^a^

Data in a same row not sharing a same superscript letter are significantly different (*p* ˂ 0.05). TFA: total fatty acid; SFA: saturated fatty acid; MUFA: monounsaturated fatty acid; n-6 PUFA: n-6 poly-unsaturated fatty acid; n-3 PUFA: n-3 polyunsaturated fatty acid.

**Table 4:** Fatty-acid compositions in the liver after fish oil-finishing (%TFA, mean ± standard error).

Fatty Acid	FO	25PO	50PO	75PO	PO
14:0	3.49 ± 0.11 ^b^	3.28 ± 0.03 ^b^	3.14 ± 0.12 ^ab^	2.79 ± 0.10 ^a^	2.84 ± 0.07 ^a^
16:0	20.06 ± 0.20	19.44 ± 0.14	20.12 ± 0.30	20.10 ± 0.11	19.53 ± 0.21
18:0	9.52 ± 0.39	9.55 ± 0.16	9.72 ± 0.20	10.35 ± 0.37	9.35 ± 0.64
20:0	0.55 ± 0.01 ^c^	0.50 ± 0.01 ^bc^	0.46 ± 0.01 ^ab^	0.47 ± 0.02 ^abc^	0.40 ± 0.03 ^a^
∑SFA	33.63 ± 0.47	32.76 ± 0.13	33.44 ± 0.42	33.71 ± 0.53	32.13 ± 0.77
16:1n-7	8.19 ± 0.21	8.02 ± 0.09	7.73 ± 0.21	7.32 ± 0.35	7.69 ± 0.11
18:1n-9	23.86 ± 0.39 ^a^	25.22 ± 0.37 ^ab^	27.04 ± 0.23 ^bc^	28.54 ± 0.76 ^cd^	29.94 ± 0.64 ^d^
20:1n-9	1.95 ± 0.10	2.02 ± 0.04	1.74 ± 0.13	1.66 ± 0.06	1.85 ± 0.05
22:1n-9	0.24 ± 0.01 ^b^	0.23 ± 0.00 ^b^	0.21 ± 0.01 ^ab^	0.22 ± 0.01 ^b^	0.19 ± 0.01 ^a^
∑MUFA	34.24 ± 0.23 ^a^	35.49 ± 0.29 ^ab^	36.73 ± 0.55 ^bc^	37.74 ± 0.52 ^cd^	39.67 ± 0.63 ^d^
18:2n-6	11.89 ± 0.18	12.16 ± 0.06	11.96 ± 0.09	11.85 ± 0.11	12.22 ± 0.33
18:3n-6	0.18 ± 0.01 ^c^	0.18 ± 0.00 ^bc^	0.16 ± 0.01 ^ab^	0.15 ± 0.00 ^a^	0.16 ± 0.00 ^ab^
20:2n-6	0.76 ± 0.02	0.81 ± 0.01	0.77 ± 0.01	0.79 ± 0.03	0.76 ± 0.01
20:4n-6	0.13 ± 0.01 ^b^	0.12 ± 0.01 ^ab^	0.10 ± 0.01 ^ab^	0.10 ± 0.00 ^ab^	0.09 ± 0.01 ^a^
∑n-6 PUFA	12.96 ± 0.19	13.27 ± 0.06	12.99 ± 0.09	12.90 ± 0.14	13.23 ± 0.33
20:3n-3	0.61 ± 0.01	0.61 ± 0.03	0.55 ± 0.01	0.54 ± 0.01	0.55 ± 0.03
20:5n-3	5.75 ± 0.10 ^c^	5.56 ± 0.07 ^bc^	5.08 ± 0.21 ^ab^	4.64 ± 0.05 ^a^	4.72 ± 0.17 ^a^
22:5n-3	4.23 ± 0.17 ^b^	4.15 ± 0.07 ^ab^	3.88 ± 0.20 ^ab^	3.72 ± 0.09 ^ab^	3.55 ± 0.09 ^a^
22:6n-3	6.92 ± 0.22 ^c^	6.60 ± 0.16 ^c^	5.89 ± 0.06 ^b^	5.32 ± 0.11 ^ab^	5.07 ± 0.10 ^a^
∑n-3 PUFA	17.51 ± 0.49 ^c^	16.92 ± 0.14 ^c^	15.40 ± 0.14 ^b^	14.22 ± 0.08 ^ab^	13.88 ± 0.25 ^a^
∑n-3/∑n-6	1.35 ± 0.02 ^d^	1.28 ± 0.00 ^c^	1.19 ± 0.01 ^b^	1.10 ± 0.01 ^a^	1.05 ± 0.02 ^a^

Data in a same row not sharing a same superscript letter are significantly different (*p* ˂ 0.05). TFA: total fatty acid; SFA: saturated fatty acid; MUFA: monounsaturated fatty acid; n-6 PUFA: n-6 poly-unsaturated fatty acid; n-3 PUFA: n-3 polyunsaturated fatty acid.

**Table 5:** Formulation and proximate composition of the experimental diets (% dry matter basis).

Ingredients	FO	25PO	50PO	75PO	PO
Fish meal	42	42	42	42	42
Corn gluten meal	8	8	8	8	8
Soybean meal	14	14	14	14	14
Wheat meal	20.68	20.68	20.68	20.68	20.68
Brewer’s yeast	5	5	5	5	5
Mineral premix ^1^	0.5	0.5	0.5	0.5	0.5
Vitamin premix ^1^	1	1	1	1	1
Monocalcium phosphate	1	1	1	1	1
L-ascorbyl-2-polyphosphate	0.2	0.2	0.2	0.2	0.2
Choline chloride	0.2	0.2	0.2	0.2	0.2
Betaine	0.3	0.3	0.3	0.3	0.3
Ethoxyquin	0.02	0.02	0.02	0.02	0.02
Mold inhibitor ^2^	0.1	0.1	0.1	0.1	0.1
Soya lecithin	1	1	1	1	1
Fish oil ^3^	6	4.5	3	1.5	0
Poultry oil	0	1.5	3	4.5	6
Proximate composition					
Crude protein	45.40	45.84	45.65	46.26	46.23
Crude lipid	9.31	10.06	9.99	10.08	9.89
Ash	9.41	9.47	9.56	9.56	9.47

^1^ Mineral premix and vitamin premix, designed for marine fish, were purchased from Qingdao Master Biotech Co., Ltd., Qingdao, China. ^2^ Contained 50% calcium propionic acid and 50% fumaric acid. ^3^ Fish oil, purchased from Qingdao Surgreen Bioengineering Co., Ltd., is on-board processed anchovy oil.

**Table 6:** Fatty acid composition of fish oil, poultry oil, and experimental diets (%TFA).

Fatty Acid	Oil		Diet				
	Fish Oil	Poultry Oil	FO	25PO	50PO	75PO	PO
14:0	5.33	0.58	5.55	4.76	4.06	3.35	2.69
16:0	18.60	26.61	21.24	22.32	22.99	23.64	24.99
18:0	4.58	5.60	4.58	4.76	4.79	4.76	4.94
∑SFA	28.51	32.79	31.37	31.84	31.84	31.75	32.62
16:1n-7	5.34	2.91	6.17	5.74	5.15	4.75	4.60
18:1n-9	16.12	44.30	15.12	18.76	22.11	25.51	29.73
20:1n-9	1.48	0.42	0.97	0.85	0.71	0.57	0.46
∑MUFA	22.94	47.63	22.25	25.35	27.97	30.84	34.80
18:2n-6	12.28	15.11	13.25	14.20	14.41	14.63	15.11
20:2n-6	0.22	0.12	0.22	0.22	0.19	0.18	0.17
20:4n-6	0.55	0.21	0.68	0.69	0.60	0.58	0.52
22:2n-6	0.38	ND	0.33	0.28	0.24	0.17	0.12
∑n-6 PUFA	13.43	15.44	14.48	15.38	15.45	15.56	15.92
18:3n-3	1.69	0.69	1.52	1.38	1.28	1.12	1.03
20:5n-3	8.15	0.06	9.66	8.38	7.24	6.07	5.11
22:5n-3	0.82	0.03	1.17	1.05	0.98	0.85	0.77
22:6n-3	8.97	0.02	7.36	6.17	5.24	4.02	2.95
∑n-3 PUFA	19.63	0.80	19.70	16.98	14.74	12.06	9.87
∑n-3/∑n-6	1.46	0.05	1.36	1.10	0.95	0.77	0.62

TFA: total fatty acid; SFA: saturated fatty acid; MUFA: mono-unsaturated fatty acid; n-6 PUFA: n-6 poly-unsaturated fatty acid; n-3 PUFA: n-3 polyunsaturated fatty acid; ND: nondetectable.

## Data Availability

The data that support the findings of this study are included in the text and in the tables. Raw data for all figures are available from the corresponding author upon reasonable request.

## Supplementary Materials

The following supporting information can be downloaded at: https://www.mdpi.com/article/10.3390/md21020122/s1, Table S1: Volatile flavor compounds detected in the muscle; Figure S1: Principal component analysis (PCA) of response values in the analysis with electronic nose; Figure S2: Linear discriminant analysis (LDA) of response values in the analysis with electronic nose.
