# Supplementary material for: Recovery of Fatty Acid and Volatile Flavor Compound Composition in Farmed Tiger Puffer (Takifugu rubripes) with a Fish Oil-Finishing Strategy
Source: Mar Drugs. 2023 Feb 13;21(2):122. doi: 10.3390/md21020122 (PMC9959805; doi:10.3390/md21020122)
Supplement: Supplementary file 1 [file marinedrugs-21-00122-s001.zip › marinedrugs-2163089-supplementary.pdf]

Table S1 Volatile flavor compounds detected in the muscle

Compound	CAS number	Formula	Relative molecule mass	Retention index	Retention time (s)	Normalized drift time	Average peak area				Flavor description
							FO-G	PO-G	FO-F	PO-F	
1	*	*	*	533.1	118.347	0.95788	106.82	159.40	169.69	181.98	
2	*	*	*	515.8	113.690	1.15655	388.63	295.70	261.49	241.23	
3	*	*	*	507.7	111.498	1.18227	240.25	263.02	329.49	244.31	
4	*	*	*	599.1	136.154	1.29339	437.65	372.84	568.55	373.52	
5	*	*	*	692.7	161.956	1.21725	178.40	220.62	208.47	267.71	
6	*	*	*	697.5	163.907	1.39281	644.14	598.10	641.70	502.03	
7	*	*	*	752.8	186.329	1.31074	70.00	40.63	59.50	19.42	
8	*	*	*	764.8	191.200	1.46707	102.63	91.84	86.88	79.24	
9	*	*	*	809.4	212.579	1.11349	862.08	559.64	704.56	699.02	
10	*	*	*	796.3	205.543	1.44713	1878.05	894.77	1190.06	891.42	
11	*	*	*	796.3	205.543	1.50379	857.67	720.25	791.00	615.56	
12	*	*	*	839.2	228.546	1.13447	387.43	168.84	178.41	193.38	
13	*	*	*	839.7	228.817	1.53107	236.27	45.67	63.27	38.70	
14	*	*	*	825.6	221.239	1.15965	68.44	83.53	33.29	33.51	
15	*	*	*	850.3	234.500	1.54576	125.06	39.85	50.65	19.19	
16	*	*	*	869.5	244.783	1.22995	49.72	48.59	27.27	24.10	
17	*	*	*	877	248.800	1.11836	123.92	73.24	89.92	66.86	

18	*	*	*	945.8	302.630	1.26479	529.75	263.18	311.89	232.97
19	*	*	*	953.1	308.857	1.345	281.31	265.31	79.88	58.91
20	*	*	*	954	309.686	1.74832	33.85	28.63	20.76	16.40
21	*	*	*	975.6	328.085	1.1044	1146.98	580.39	701.25	597.52
22	*	*	*	989.2	339.685	1.4167	1439.37	1505.30	1350.99	754.68
23	*	*	*	959.2	314.086	1.56641	35.38	27.23	19.10	14.44
24	*	*	*	536.3	119.207	1.21169	121.26	74.49	57.22	61.49
hexanal monomer	C66251	C6H12O	100.2	798.3	206.593	1.25750	1443.08	1670.85	1621.18	1633.92
hexanal dimer	C66251	C6H12O	100.2	797.4	206.119	1.56335	3371.09	4451.16	4020.98	3215.95
Heptanal monomer	C111717	C7H14O	114.2	903.3	266.386	1.33578	1529.24	1398.13	1679.97	1223.29
Heptanal dimer	C111717	C7H14O	114.2	902.9	266.043	1.69764	1076.85	758.68	1154.96	489.73
2-pentenal (E) monomer	C1576870	C5H8O	84.1	751.4	185.745	1.10570	564.71	424.45	507.38	263.01
2-pentenal (E) dimer	C1576870	C5H8O	84.1	751.7	185.878	1.36295	436.24	168.20	252.15	59.96
Octanal monomer	C124130	C8H16O	128.2	1006.8	360.810	1.40319	2153.71	1724.51	2184.78	1193.04
Octanal dimer	C124130	C8H16O	128.2	1006.5	360.279	1.82740	498.51	310.41	487.06	172.83
2-Ethyl-1-hexanol	C104767	C8H18O	130.2	1030.6	394.950	1.41081	330.81	267.16	169.00	104.21
Nonanal monomer	C124196	C9H18O	142.2	1111.1	510.795	1.47572	3096.13	2742.43	2900.36	2106.80
Nonanal dimer	C124196	C9H18O	142.2	1110.1	509.264	1.94344	428.21	377.09	357.33	241.42
1-butanol	C71363	C4H10O	74.1	668.2	154.775	1.37675	972.73	332.08	446.13	388.89
2-Butanone monomer	C78933	C4H8O	72.1	591.1	133.993	1.05998	882.58	1045.07	939.92	1137.84
2-Butanone dimer	C78933	C4H8O	72.1	592.8	134.448	1.24806	1434.57	1395.76	1169.98	1254.01
ethyl acetate monomer	C141786	C4H8O2	88.1	612.2	139.688	1.09847	291.90	293.25	476.52	340.97

ethyl acetate dimer	C141786	C4H8O2	88.1	609.7	139.005	1.33825	86.32	84.25	222.21	121.58
1-pentanol monomer	C71410	C5H12O	88.1	764.2	190.947	1.24696	392.27	437.98	430.58	304.64
1-pentanol dimer	C71410	C5H12O	88.1	763.1	190.515	1.50913	36.82	44.92	38.70	22.07
Benzaldehyde monomer	C100527	C7H6O	106.1	961.1	315.685	1.15486	774.78	796.97	585.02	581.17
Benzaldehyde dimer	C100527	C7H6O	106.1	959.9	314.665	1.47145	187.31	194.54	100.44	109.17
1-Octen-3-ol monomer	C3391864	C8H16O	128.2	983.2	334.568	1.16086	607.19	489.41	488.18	357.04
1-Octen-3-ol dimer	C3391864	C8H16O	128.2	981.6	333.231	1.60055	72.93	49.34	50.38	41.04
methyl acetate monomer	C79209	C3H6O2	74.1	533.2	118.379	1.03286	504.61	463.75	363.08	462.07
methyl acetate dimer	C79209	C3H6O2	74.1	533.5	118.466	1.19366	152.64	120.20	94.99	108.28
Ethanol	C64175	C2H6O	46.1	467.7	100.721	1.04601	1155.10	1458.88	670.98	1824.88
Acetone	C67641	C3H6O	58.1	509.1	111.887	1.11790	841.36	827.05	886.95	942.99
1-propanol	C71238	C3H8O	60.1	555.8	124.465	1.11001	2351.74	1907.09	1868.97	1650.91
1-penten-3-ol	C616251	C5H10O	86.1	698.9	164.464	0.94049	4028.26	4021.47	3952.61	3241.32
3-methylbutanal	C590863	C5H10O	86.1	659.6	152.480	1.40314	501.28	307.85	289.36	253.83
3-Pentanone	C96220	C5H10O	86.1	696.1	163.349	1.35873	1031.99	664.06	755.89	588.07
Pentanal	C110623	C5H10O	86.1	698.9	164.464	1.41966	515.87	578.55	636.68	419.04
3-methyl-2-butenal monomer	C107868	C5H8O	84.1	740.8	181.465	1.08920	178.73	219.42	109.43	107.41
3-Methyl-2-butenal dimer	C107868	C5H8O	84.1	738.8	180.629	1.35460	71.76	59.22	28.11	12.39
(E)-2-hexenal monomer	C6728263	C6H10O	98.1	850.3	234.500	1.18379	1077.66	593.50	858.37	429.22
(E)-2-Hexenal dimer	C6728263	C6H10O	98.1	849.8	234.229	1.52163	160.67	57.55	116.41	25.24
n-Hexanol	C111273	C6H14O	102.2	870.3	245.218	1.32654	64.23	62.12	57.23	35.58
4-heptenal (Z)	C6728310	C7H12O	112.2	899.8	263.432	1.14948	1295.21	784.74	1154.53	609.55

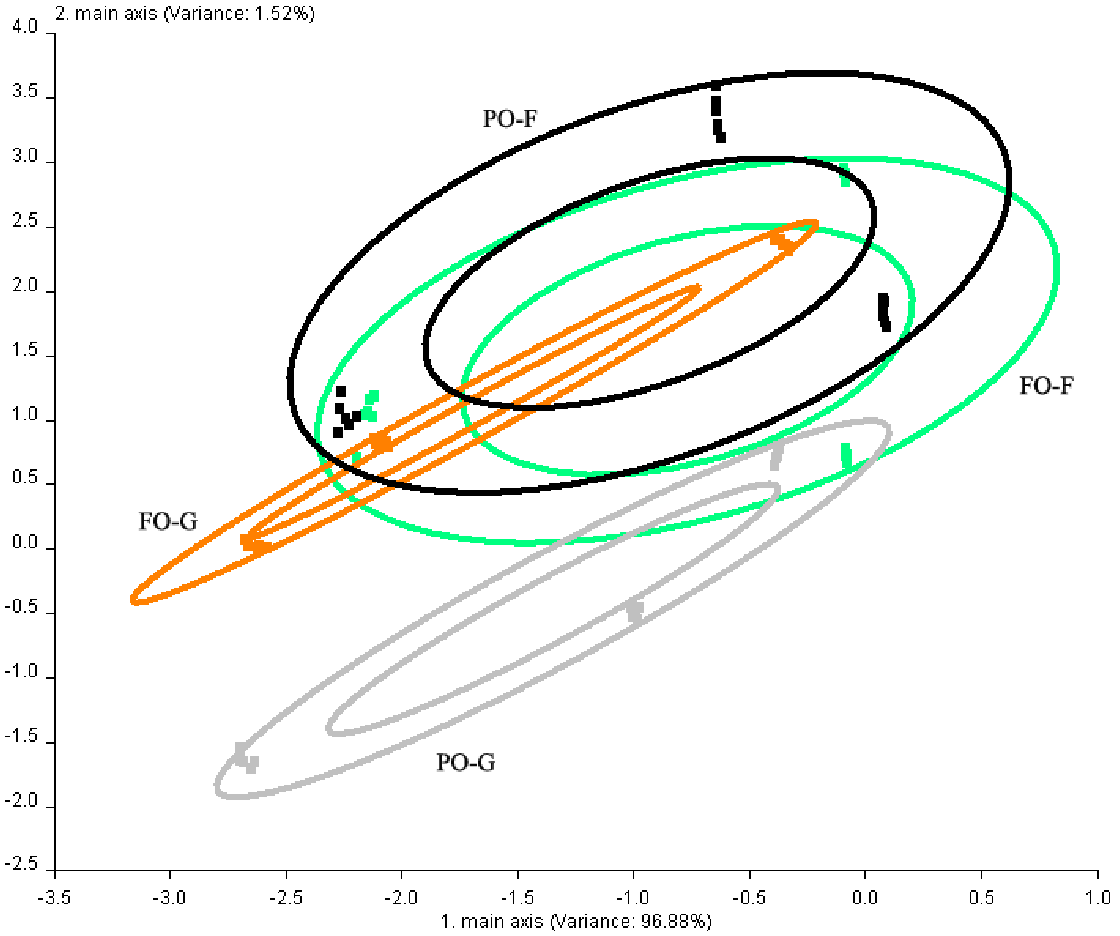

Figure S1 Principal Component Analysis (PCA) of response values in the analysis with electronic nose. FO-G and PO-G: The fish oil and poultry oil group at the end of growing-out period, respectively; FO-F and PO-F: The fish oil and poultry oil group after the fish oil-finishing period, respectively. Small distance indicates high similarity, while far apart samples have significant differences.

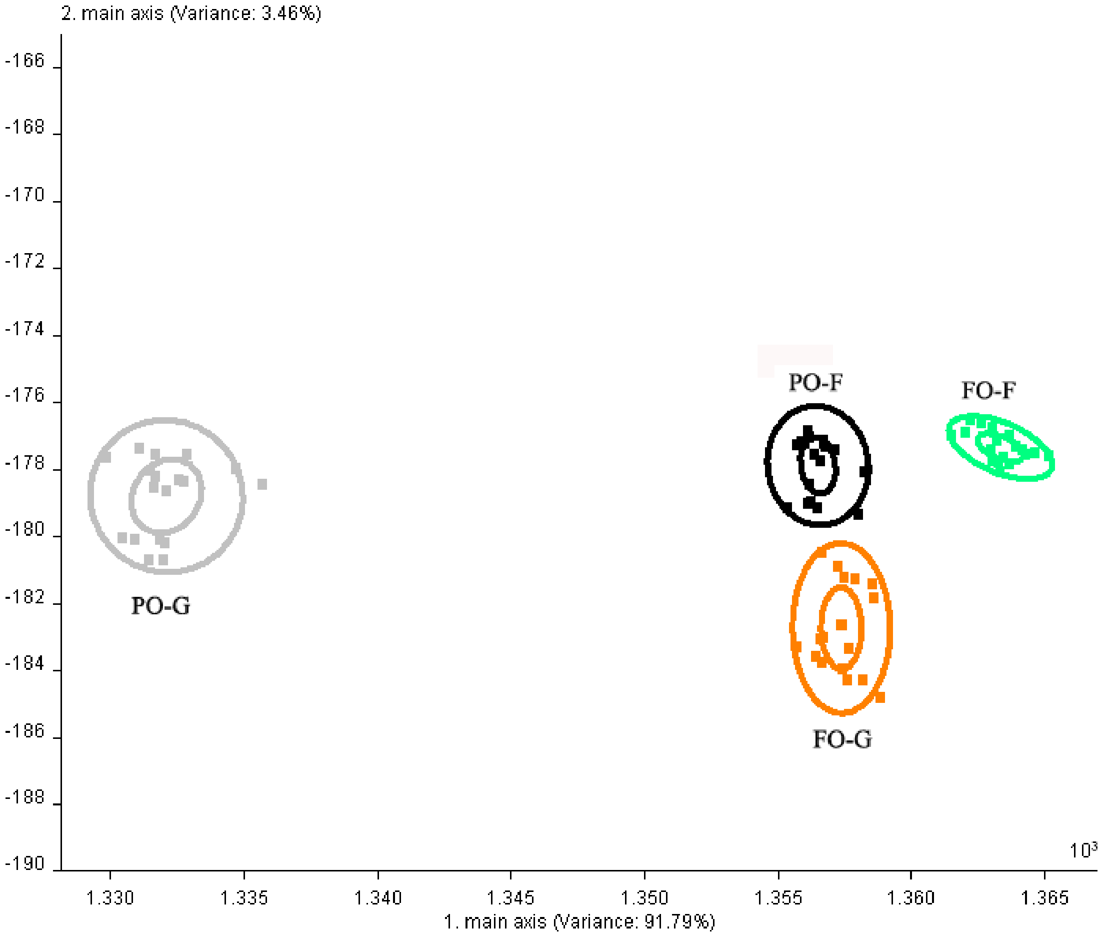

Figure S2 Linear Discriminant Analysis (LDA) of response values in the analysis with electronic nose. FO-G and PO-G: The fish oil and poultry oil group at the end of growing-out period, respectively; FO-F and PO-F: The fish oil and poultry oil group after the fish oil-finishing period, respectively. LDA is aimed at selecting the projection direction with the best classification performance for sample data through the algorithm, so that each group of data can be separated as far as possible.
